# The Stenographic Art

**Published:** 1890-08

**Authors:** 


### THE STENOGRAPHIC ART.

Dickens, in one of those inimitable retrospects, that halt along the course to take a backward look, in David Copperfield, and which are said to reflect something of his own life, remarks ; " I have tamed that savage stenographic mystery. I make a respectable income by it. I am in high repute for my accomplishment in all pertaining to the art." * * * " I wallow in words." And what a chaos of quirks and slants and pothooks, dots and dashes, in every conceivable state of apparent disorder, my pretty cousin shows me in her little oblong book as she sits down to decipher them, after attending a meeting of the Chatterbox Club in her official character of reporter. I look over her shoulder as she writes, and wonder that brain and muscle could ever be trained to disentangle such a conglomerated mass, worse even than the hieroglyphics of the Egyptian Obelisk that is trying to get acclimated in Central Park.

But David Copperfield was written when such a personage as a "female reporter" had not dawned upon the world. Woman in that day was a comparatively helpless member of society. The most that could be expected of her in relation to reporting, was to sit by and hold her lord's pens, as Dicken's young and pretty wife used to do in his hours of mental struggle with his hieroglyphical abstractions. Now, in these latter days all this is changed, and woman's quicker brain and nimbler fingers have fairly ran away with this " savage stenographic mystery," and her salaried employment in halls of State, public assemblies and business centres, in short everywhere that " short hand " is resorted to, is a common thing. Indeed, she is quicker to learn and readier to practice her art of capturing syllables as they flow, and crystalizing them into understandable shapes than her male competitor, and this is only one of the many arts she has mastered which were formerly deemed unsuited to her womanly nature. Verily she leads the way where once she was led, and forcefully wields the pen she was wont to sit idly by and hold, unused, and the home fireside is all the brighter for it.

---

WHITE RIBBONS cannot be washed in water to look well, but in gasoline they show no trace of ever being used before. Lay them to soak in gasoline, covering the dish for a few minutes, then wash thoroughly and lay for a few minutes again in clean gasoline, to rinse. Do not wring, but hang in the open air. When dry, fold and lay in a book under a heavy weight. When they no longer look clear they can be colored with diamond dyes, but can never be made to look new after having once been washed and ironed.

